# Supplementary material for: A Multiepitope Nanovaccine Candidate Adjuvanted with Porcine Ferritin Scaffold for African Swine Fever Virus
Source: Vaccines (Basel). 2025 May 30;13(6):585. doi: 10.3390/vaccines13060585 (PMC12197706; doi:10.3390/vaccines13060585)
Supplement: Supplementary file 1 [file vaccines-13-00585-s001.zip › vaccines-3594312-supplementary.pdf]

Table. S1 12 strains of ASFV that have been prevalent in the past decade.

NCBI RefSeq	Genome size	Strain	Date	Submitter
GCF_000858485.1	170.1 kb	BA71V	2014	Centro de Biologia Molecular Sebero Ochoa
GCF_003032865.1	182.4 kb	L60	2019	Biochemistry and Microbiology, University of Victoria
GCF_003032875.1	180.4 kb	BA71	2019	Centro de Biologia Molecular Severo Ochoa (CSIC-UAM)
GCF_003032885.1	172.1 kb	NHV	2019	Biochemistry and Microbiology, University of Victoria
GCF_003032905.1	191.1 kb	Ken05/Tk1	2019	Biochemistry and Microbiology, University of Victoria
GCF_003032915.1	184.4 kb	Ken06.Bus	2019	Biochemistry and Microbiology, University of Victoria
GCF_003032925.1	182.9 kb	26544/OG10	2019	Istituto Zooprofilattico Sperimentale della Sardegna "G. Pegreffi"
GCF_003033005.1	184.6 kb	47/Ss/2008	2019	Department of Biomedical Sciences and Veterinary Public Health, Swedish University of Agricultural Sciences (SLU)
GCF_003047675.1	182.3 kb	Benin 97/1	2019	Chapman D.A, Microbiology, Institute of Animal Health, Pirbright Laboratory, Ash Road, Pirbright, Woking, Surrey, GU24 0NF, UNITED KINGDOM
GCF_003047695.1	171.7 kb	OURT 88/3	2019	Chapman D.A, Microbiology, Institute of Animal Health, Pirbright Laboratory, Ash Road, Pirbright, Woking, Surrey, GU24 0NF, UNITED KINGDOM
GCF_003047715.1	181.2 kb	E75	2019	De Villiers E.P, International Livestock Research Institute, Naivasha Road, PO Box 30709, Nairobi, 00100, KENYA
GCF_003047755.2	190.6 kb	Georgia 2007/1	2020	Friedrich-Loeffler-Institute, Federal Research Institute for Animal Health, Institute of Diagnostic Virology, Suedufer 10, Greifswald - Insel Riems, Mecklenburg - Western Pomerania, 17493, Germany

Table. S2 105 relatively conserved proteins with >90% amino acid sequence homology

ORF	Gene description	Gene expression
KP86R	putative transmembrane protein	unknown
KP93L	putative transmembrane protein	unknown
KP177R	membrane protein; member of the multigene family p22; contains N-terminal transmembrane region	early
KP360L	member of multigene family 360; contains helix-turn-helix motif	early
MGF 360-1L	member of multigene family 360; contains helix-turn-helix motif	early
DP363R	member of multigene family 360	early
MGF 360-19R	member of multigene family 360; (DP363R)	early
DP96R	putative	unknown
DP71L	putative product is similar to mouse protein MyD116	unknown
MGF 360-18R	member of multigene family 360 (DP148R)	unknown
MGF 505-11L	member of multigene family 360 (DP542L)	unknown
L11L	similar to African swine fever virus E75 strain E75 of INSD accession FN557520	unknown
MGF 360-16R	similar to African swine fever virus E75 strain E75 of INSD accession FN557520	unknown
I215L	unknown	immediate-early
G1211R	DNA polymerase alpha-like protein	unknown
CP2475L	220 kDa polyprotein	unknown
CP204L	ASFV-induced protein p3	unknown
CP530R	60 kDa polyprotein	unknown
NP1450L	RNA polymerase subunit 1	unknown
D339L	RNA polymerase II subunit 7 (RPB7)	unknown
D1133L	member of helicase superfamily II group	unknown
D345L	hypothetical protein	unknown
P1192R	topoisomerase I9	unknown
H359L	RNA polymerase subunit 3	unknown
E423R	hypothetical protein	unknown
Q706L	helicase	unknown
E248R	superfamily of myristoylated proteins	unknown
I329L	inhibitor of the Toll-like receptor 3 (TLR3) signaling pathway	unknown
G1340L	putative product contains ATP- or GTP-binding motif; similar to poxvirus early transcription factor (VETF), large subunit; putative transcription factor	unknown
B646L	similar to Chlorella virus PBCV-1 major capsid protein; similar to Tipoula iridescent virus type 1 major capsid protein	late
B602L	product contains highly variable tandem repeats; cysteine-rich region similar to that of the epidermal growth factor receptor	late

	required for the correct folding of the capsid protein p72	
B475L	putative product contains three overlapping leucine zippers; contains tandem repeats; glutamic acid-rich	unknown
B438L	product contains cell attachment sequence RGD; contains helix-turn-helix motif; structural protein p49, required for formation of the vertices in icosahedral capsid	unknown
B962L	member of helicase superfamily II group including 'DEAH' pre-mRNA processing proteins and vaccinia I8R	late
C962R	putative product contains ATP- or GTP-binding motif; putative DNA primase	unknown
C475L	similar to the poly(A) polymerases of members of the family of nucleocytoplasmic large DNA viruses; putative poly(A) polymerase	unknown
p110-9L	member of Multigene Family 110 (Lis 290)	unknown
MGF 110-9L	member of Multigene Family 110 (Lis 290)	unknown
MGF 110-11L	member of multigene family 110 (Lis 119_1)	unknown
MGF 110-13L	member of Multigene Family 110 (Lis117)	unknown
MGF 110-14L	member of Multigene Family 110 (Lis 121_2)	unknown
LIS117	viral family 110; pfam01639	unknown
MGF 360-6L	member of multigene family 360 (LIS375)	unknown
MGF_360-13L	member of multigene family 360 (360-1)	unknown
MGF_505-2R	member of multigene family 505 (A489R)	late
p505 2R	multigene family 530 protein; pfam03158	unknown
K205R	unknown	early
M1249L	VOCs Family: Unknown (BA71V-M1249L)	unknown
M448R	putative product contains microbody targeting signal; similar to the T4 RNA ligase 1 (Rnl1) family of RNA ligases	unknown
EP364R	similar to ERCC4-type DNA repair nuclease	unknown
EP402R	membrane protein; responsible for hemadsorption of ASFV-infected cells; nonessential; contains N-terminal hydrophobic segment and central transmembrane region	late
EP424R	contains FTS J-like methyl transferase domain; putative methyltransferase	unknown
EP1242L	RNA polymerase subunit 2	late
replication	member of helicase superfamily II; similar to herpesvirus origin-binding protein encoded by GenBank Accession Number M68963	unknown
F1055L	member of helicase superfamily II; similar to herpesvirus origin-binding protein encoded by GenBank Accession Number M68963	unknown
A859L	member of helicase superfamily II group including hsdR subunits of type Ib restriction enzymes; contains two overlapping leucine zippers	unknown
5EL	similar to African swine fever virus E75 strain E75 of INSD	unknown

	accession FN557520	
A238L	putative product contains IkB-like ankyrin repeats; non-essential	unknown
MGF_360-15R	member of multigene family 360 (A276R)	unknown
A276R	member of multigene family 360	unknown
A151R	contains CXXC motif; interacts with pB119L and pE248R; 'component of the viral system for disulfide bond formation	early
BA71-A240L	thymidylate kinase	early
MGF 505-3R	member of multigene family 505 (A280R); contains internal 6-aa repeat	early
A280R	member of multigene family 505; contains internal 6-aa repeat	early
BA71-A489R	member of multigene family 505	late
MGF 360-12L	member of multigene family 360 (pKP360L)	unknown
MGF 505-1R	member of multigene family 505 (pA489R)	unknown
MGF 360-9L	member of multigene family 360 (pL356L)	unknown
J319L	member of multigene family 360	early
MGF 360-8L	member of multigene family 360 (J319L)	early
F317L	VOCs Family: Unknown (BA71V-F317L)	unknown
H124R	VOCs Family: Unknown (BA71V-H124R)	unknown
I8L	similar to African swine fever virus E75 strain E75 of INSD accession FN557520	unknown
I9R	VOCs Family: Unknown (Malawi-19R)	unknown
I73R	unknown	early
M448R	putative product contains microbody targeting signal; similar to the T4 RNA ligase 1 (Rnl1) family of RNA ligases	unknown
QP383R	NifS-like protein;similar to pyridoxal phosphate-dependent aminotransferases of class V	unknown
E184L	putative	unknown
S183L	putative	unknown
A151R	contains CXXC motif; interacts with pB119L and pE248R; gene expression early 'component of the viral system for disulfide bond formation	unknown
A224L	IAP homolog; putative product contains BIR motif of baculovirus IAP proteins; cysteine-rich	unknown
D205R	RNA polymerase subunit 5; putative	unknown
I329L	inhibitor of the Toll-like receptor 3 (TLR3) signaling pathway	late
K145R	unknown	late
K205R	unknown	early
MGF-110-1L	member of multigene family 110 (L270L); putative transmembrane protein;	immediate-early
MGF-360-14L	member of multigene family 360 (360-2); hypothetical protein	unknown
MGF-360-15R	member of multigene family 360 (A276R); hypothetical protein	unknown
MGF 505-4R	member of multigene family 505 (A505R), DUF249; Multigene family 530 protein, these proteins may be involved in	early

	promoting survival of infected macrophages.	
MGF 505-7R	DUF249; Multigene family 530 protein, these proteins may be involved in promoting survival of infected macrophages.	unknown
MGF 505-8R	member of Multigene Family 505 (A528-2R)	unknown
pBA71-A469R	member of multigene family 505	unknown
MGF 360-1L	Member of Multigene Family 360 (KP360L); contains helix-turn-helix motif	early
MGF 360-2L	Member of Multigene Family 360 (KP362L)	early
MGF 360-4L	ASFV_360; African swine fever virus multigene family 360 protein	unknown
MGF 360-5L	unknown	unknown
MGF-360-15R	member of multigene family 360 (A276R); hypothetical protein	unknown
pJ268L	member of multigene family 300	early
MGF 300-4L	member of multigene family 300 (J182L)	unknown
C84L	unknown, but C84L gene affects the virulence of the SY18 strain	unknown
C717R	unknown	unknown
MGF_110-5L	member of multigene family 110 (V82L)	early
MGF-110-6L	v110; Viral family 110	unknown
MGF-110-7L	v110; Viral family 110	unknown
MGF-110-8L	v110; Viral family 110	unknown
pJ328L	member of multigene family 300	early
pA505R	member of multigene family 505	early
MGF-530-2R	DUF249; Multigene family 530 protein	unknown
pBA71-L270L	member of multigene family 110; putative transmembrane protein; contains two family 110 domains; contains two central transmembrane regions	immediate-early

Table. S3 Epitope composition of T-cell epitope protein MEP1

ORF	Total number of T-cell epitopes	Selected epitope	Position(aa)	Score	Percentile-rank
KP86R	438	NQHIIGDSY**	40-48	0.591559	0.11
KP93L	5	TPSNNTVPPPY**	54-64	0.545648	0.12
KP177R	16	NPHHPVLKY	14-22	0.97566	0.01
KP360L	36	NENKILEIF	44-52	0.502449	0.2
MGF 360-1L	36	ISFNEMLTRYW	33-43	0.502449	0.2
DP363R	2121	HEAPIILCF	41-49	0.799712	0.01
MGF 360-19R	1659	WHEAPIILCF	40-49	0.799712	0.01
DP96R	532	TSSEWIAEY**	47-55	0.840481	0.02
DP71L	420	RFAAAVEVW	18-26	0.691396	0.05
MGF 360-18R	1498	CPLDILTGY	10-18	0.878409	0.01
MGF 505-11L	3234	SVYYSIIKY	39-47	0.890927	0.01
L11L	420	VMAPIPLVL	7-15	0.655145	0.02
MGF 360-16R	1253	YFDNHIPNI**	29-37	0.805651	0.01
I215L	1260	VAFPPEYPY	54-62	0.862264	0.01

Note: *Partial overlap between T-cell epitopes and B cell epitopes; **complete overlap between T-cell epitopes and B cell epitopes.

Table. S4 Epitope composition of T-cell epitope protein MEP2

Protein	Total number of T-cell epitopes	Selected epitope	Position(aa)	Score	Percentile-rank
G1211R	7019	AADDTTCYY*	7-15	0.983519	0.01
		MPIDIHEVRY*	35-44	0.942423	0.01
CP2475L	14321	HIDKNIIQY**	32-40	0.996633	0.01
CP204L	1093	VVFHAGSLY*	17-25	0.826497	0.03
CP530R	3151	YSDPETVHSY*	23-32	0.987543	0.01
NP1450L	8681	ILDLIRLQY*	42-50	0.986404	0.01
D339L	2024	SVYHVQEEL*	22-30	0.668878	0.01
D1133L	6742	VPAKPEHLY*	7-15	0.975829	0.01
D345L	2066	HIDGTYLGY*	22-30	0.990783	0.01
P1192R	7141	MPVYQELGY*	58-66	0.911787	0.01
H359L	12917	IPDISFVGY*	22-30	0.900678	0.01
E423R	2521	SEYKQYNEF**	31-39	0.743031	0.01
Q706L	4201	IVDEAHNLF*	26-34	0.988011	0.01
E248R	1457	FIADAISAV*	48-56	0.657875	0.02
I329L	1954	ISFSNNNTY*	40-48	0.867971	0.01

Note: *Partial overlap between T-cell epitopes and B cell epitopes; **complete overlap between T-cell epitopes and B cell epitopes.

Table. S5 Epitope composition of T-cell epitope protein MEP3

Protein	Total number of T-cell epitopes	Selected epitope	Position(aa)	Score	Percentile-rank
G1340L	7986	SPLQMEKQY	47-55	0.923431	0.01
		SSMSVSTFW	41-49	0.902767	0.01
B646L	3822	ISDISPVTY**	32-40	0.989685	0.01
B602L	3346	KVDEFYYKY**	54-62	0.99473	0.01
B475L	2785	AIDQDKPEF**	46-54	0.979332	0.01
B438L	2395	SEHTKFFSY	30-38	0.829441	0.01
B962L	5754	IPKNVVQTY	55-63	0.930857	0.01
		KPDGFLIVY	51-59	0.905653	0.01
C962R	5754	QPHETNILNY**	4-13	0.89872	0.01
		SVLEKYLQW*	47-55	0.9017	0.01
C475L	2835	KTDFNVSKY**	6-14	0.99426	0.01
		KTEFPQPSY**	27-35	0.98027	0.01
		YPALVLATY	54-62	0.92457	0.01

Note: *Partial overlap between T-cell epitopes and B cell epitopes; **complete overlap between T-cell epitopes and B cell epitopes.

Table. S6 Epitope composition of T-cell epitope protein MEP4

Protein	Total number of T-cell epitopes	Selected epitope	Position(aa)	Score	Percentile-rank
p110-9L	25	HPTIIENNY**	21-29	0.861539	0.01
MGF 110-9L	20	YYDWFDELM**	40-48	0.736237	0.01
		YEAHFRIHY	44-52	0.733234	0.02
MGF 110-11L	13	KVWDDMSSV**	43-51	0.5461	0.03
MGF 110-13L	13	KVWDDMSSV**	43-51	0.5461	0.03
MGF 110-14L	13	HPPKSELSY*	28-36	0.814951	0.03
		ISYKMPNHF*	19-27	0.841051	0.02
LIS117	19	YFIEPKIPY*	24-32	0.735323	0.01
		MPLIVQNDY**	1-9	0.850326	0.02
MGF 360-6L	45	NVDEIHHAY*	13-31	0.994366	0.01
		STYEYTETF**	13-21	0.929575	0.01
MGF_360-13L	31	SIDEHCILKY*	21-30	0.983692	0.01
		SLIKLFTEW	6-14	0.834337	0.01
MGF_505-2R	52	YLHETLFEL	20-28	0.811301	0.01
		QYDLIHKY	23-30	0.764837	0.01

Note: *Partial overlap between T-cell epitopes and B cell epitopes; **complete overlap between T-cell epitopes and B cell epitopes.

Table. S7 Epitope composition of T-cell epitope protein MEP5

Protein	Total number of T-cell epitopes	Selected epitope	Position(aa)	Score	Percentile-rank
p505 2R	54	YLHETLFEL	20-28	0.811301	0.01
K205R	18	SEWASSKTF**	5-13	0.803438	0.01
		SFENFIERY*	40-48	0.787068	0.01
M1249L	7483	KADSSLNAY*	53-61	0.986911	0.01
		VTDPASALLY*	11-20	0.981008	0.01
		AVDSAVRIF	18-26	0.975501	0.01
M448R	2646	SQVPELPQY**	58-66	0.87216	0.01
		SFPETLENL*	5-13	0.83012	0.01
EP364R	2163	SQKLVQLFY*	2-10	0.750218	0.01
		HPTIIENNY	21-29	0.861539	0.01
EP402R	2009	SVDSPTITY*	38-46	0.999264	0.01
		KSVDSPTITY*	37-46	0.995025	0.01
EP424R	2520	TPLVDLSLY*	56-64	0.890398	0.01

Note: *Partial overlap between T-cell epitopes and B cell epitopes; **complete overlap between T-cell epitopes and B cell epitopes.

Table. S8 Epitope composition of T-cell epitope protein MEP6

Protein	Total number of T-cell epitopes	Selected epitope	Position(aa)	Score	Percentile-rank
EP1242L	2879	KSDENAVQY**	1-9	0.994987	0.01
		IVDNNIEKY*	45-53	0.991822	0.01
K205R	11	SFENFIERY*	40-48	0.787068	0.01
replication	127	KTDPQTILKF*	61-70	0.978877	0.01
		LPDTQKHIY**	23-31	0.906415	0.01
F1055L	125	KTDPQTILKF*	57-66	0.978877	0.01
		LPDTQKHIY**	19-27	0.906415	0.01
A859L	79	QTLEKLYQY*	15-23	0.797358	0.01
		ESMAFLETW	50-58	0.830789	0.01
5EL	18	YKLDVFHRW	39-47	0.832585	0.01
A238L	27	GADPTQKDY*	4-12	0.949539	0.02
		KFPEQNPNF	9-17	0.75362	0.01
MGF_360-15R	20	NTFNPVYKY*	30-38	0.888986	0.01
A276R	21	NTFNPVYKY*	30-38	0.888986	0.01
A151R	13	SYIDNSYKY*	37-45	0.907366	0.01
BA71-A240L	18	MYDDKYLNV*	61-69	0.697021	0.02

Note: *Partial overlap between T-cell epitopes and B cell epitopes; **complete overlap between T-cell epitopes and B cell epitopes.

Table. S9 Epitope composition of T-cell epitope protein MEP7

Protein	Total number of T-cell epitopes	Selected epitope	Position(aa)	Score	Percentile-rank
MGF 505-3R	31	KYHDLVYKY**	24-32	0.913601	0.01
		YQHKHILKY**	56-64	0.893515	0.01
A280R	32	KYYDLVYKY**	24-32	0.949489	0.01
		YQHKHILKY**	56-64	0.893515	0.01
BA71-A489R	53	YLHETLFEL	20-28	0.811301	0.01
MGF 360-12L	45	KTDLLNNEF*	25-33	0.983607	0.01
		NVFDLHEAY*	9-17	0.893007	0.01
MGF 505-1R	66	QAMLTSIQY	55-63	0.869912	0.01
		SLIKLFTEW	2-10	0.834337	0.01
MGF 360-9L	47	YPSIYSKHY*	31-39	0.920115	0.01
		FFDDYILQL*	20-28	0.860998	0.01
J319L	35	NVFDLHELY*	18-26	0.904783	0.01
MGF 360-8L	43	VPMNIFVKY	29-37	0.973551	0.01

Note: *Partial overlap between T-cell epitopes and B cell epitopes; **complete overlap between T-cell epitopes and B cell epitopes.

Table. S10 Epitope composition of T-cell epitope protein MEP8

Protein	Total number of T-cell epitopes	Selected epitope	Position(aa)	Score	Percentile-rank
F317L	2149	QMDKLGFLL	5-13	0.745437	0.20
		SNAHITQTM*	26-34	0.410158	0.61
H124R	798	EYVQVVQKF*	4-12	0.64083	0.09
		VVGGSKPTYW	26-35	0.433535	0.52
I8L	651	YYYGEQQNL**	15-23	0.63365	0.03
		EQQNLKQIW*	19-27	0.57957	0.07
I9R	602	KSDDAVCKYL*	11-20	0.281787	0.35
		TTYNPVVDW*	49-57	0.228559	0.52
I73R	434	MVKEALEKY*	9-17	0.724204	0.07
		IQKEHNVVL*	29-37	0.499809	0.04
M448R	2646	AIQSEVIEWL*	24-33	0.188915	1.70
		AQESLLTTM*	89-97	0.13536	1.10

Note: *Partial overlap between T-cell epitopes and B cell epitopes; **complete overlap between T-cell epitopes and B cell epitopes.

Table. S11 Epitope composition of T-cell epitope protein MEP9

Protein	Total number of T-cell epitopes	Selected epitope	Position(aa)	Score	Percentile-rank
QP383R	2612	GLYAEVPKF*	9-17	0.473743	0.12
		FYIQQILNL	34-42	0.548416	0.09
E184L	1219	RISSELISY*	23-31	0.917019	0.04
		ILNHICHQY	35-43	0.660539	0.34
S183L	1212	SVVVGGVEY*	2-10	0.742775	0.02
		YSLNNWARY**	10-18	0.70782	0.08
A151R	988	SYIDNSYKY**	37-45	0.874722	0.01
		STSVGPHIF**	117-125	0.75319	0.06
A224L	1499	SIDARNQSF**	28-36	0.984463	0.01
		MIDSYNDYY*	104-112	0.969033	0.02
D205R	1366	SASDPPVVW*	165-173	0.876692	0.01
		ITQEEAQEF**	142-150	0.810249	0.03

Note: *Partial overlap between T-cell epitopes and B cell epitopes; **complete overlap between T-cell epitopes and B cell epitopes.

Table. S12 Epitope composition of T-cell epitope protein MEP10

Protein	Total number of T-cell epitopes	Selected epitope	Position(aa)	Score	Percentile-rank
I329L	2234	RSNTPTYLY*	136-144	0.905243	0.04
		KQDKSSHNY**	61-69	0.971924	0.02
K145R	946	KLDPIGFINY**	58-67	0.984308	0.01
		SPNKYNNFY**	108-116	0.956152	0.01
K205R	1366	NIQDLQNKY**	54-62	0.822688	0.11
		MVEPREQFF*	1-9	0.908277	0.04
MGF-110-1L	1821	CSQPTHFKW*	41-49	0.799494	0.01
		TSIKPHKTY*	20-28	0.858118	0.02
MGF-360-14L	2430	VLDVTEPQEIY**	319-329	0.905836	0.04
		FVDWGANPEY*	81-90	0.952894	0.02
MGF-360-15R	1594	VVDDVPSIDY*	83-92	0.984641	0.01
		NTFNPVYKY*	113-121	0.888986	0.01

Note: *Partial overlap between T-cell epitopes and B cell epitopes; **complete overlap between T-cell epitopes and B cell epitopes
